# Association Between Vitamin D Deficiency and Tumor Characteristics in Breast Cancer Patients

**DOI:** 10.7759/cureus.62296

**Published:** 2024-06-13

**Authors:** Manish Swarnkar, Krishna Kumar, Pankaj Prasad, Kritika Singhal

**Affiliations:** 1 General Surgery, All India Institute of Medical Sciences, Bhopal, Bhopal, IND; 2 Community and Family Medicine, All India Institute of Medical Sciences, Bhopal, Bhopal, IND

**Keywords:** vitamin d deficiency, calcium levels, breast histopathology, breast metastasis, lymph node status, breast cancer biology

## Abstract

Introduction

Breast cancer is the most frequent cancer among women worldwide, but there is little literature regarding the effects of vitamin D on breast cancer patients in the Indian population. Hence, this study was planned to determine the correlation between vitamin D deficiency and tumor characteristics in breast cancer patients.

Methods

This was a cross-sectional study conducted in a tertiary healthcare facility in central India among all newly diagnosed patients with breast carcinoma who had received primary surgery and pathological confirmation. We performed universal sampling and included 50 patients in the study. We excluded patients with insufficient histopathological reports, those unfit for surgery, and those with hepatic or renal failure, metabolic bone disease, malabsorption, or recent consumption of vitamin D (patients who had received oral vitamin D in the preceding two weeks, or vitamin D injection in the preceding six months).

Results

Among the 50 patients, 86% were vitamin D deficient, with a mean deficiency of 23.54. Vitamin D deficiency is most common in the age groups 41-50 years and >60 years, with the mean age group of 51.49 years. The left side is more involved than the right in vitamin D-deficient patients. Most patients were moderately and poorly differentiated, suggesting a significant association between vitamin D deficiency and tumor differentiation.

Almost half the patients were estrogen receptor (ER), progesterone receptor (PR), and human epidermal growth factor receptor 2 (Her-2/neu) status negative with vitamin D deficiency. Vitamin D deficiency was highest in Her-2/neu amplified, luminal A, and B patients. The mean lymph node-positive participants was 4.04, and the mean number of lymph nodes extracted was 15.58 in vitamin D-deficient breast cancer patients.

Conclusion

The prevalence of low vitamin D status was high among breast cancer patients. There is an association between vitamin D deficiency and tumors with poor prognostic features. Low vitamin D levels were considered a risk factor for ER, PR, and Her-2/neu-negative tumors along with positive lymph node status in breast cancer patients. Vitamin D status is a modifiable risk factor for breast cancer. Thus, it is concluded from this study that vitamin D has a potential role in the prevention of breast cancer, it may reduce its aggressiveness, and its deficiency is associated with an increased risk of breast cancer.

## Introduction

Breast cancer is the most frequent cancer among women all over the world [1]. According to the World Health Organization (WHO), in 2022, there were 2.3 million women diagnosed with breast cancer and 670,000 deaths globally. Breast cancer occurs in every country of the world in women at any age after puberty but with increasing rates in later life. Various prognostic variables, most notably lymph node stage, tumor size, and histological grade, are used to guide the clinical management of this malignancy [2]. Vitamin D insufficiency has been linked to an increased risk of breast, prostate, and colon cancers [1]. It is thought that low vitamin D levels cause unrestricted cellular proliferation, angiogenesis, and metastasis [3]. Vitamin D slows cell proliferation, causes differentiation and death, and has antiangiogenesis actions in both normal and malignant breast cells, according to numerous preclinical investigations [4]. Breast cancers in patients with low 25(OH)D levels (below 30 or 32 ng/mL) are more aggressive clinicopathologically, which results in a worse prognosis [5]. The circulating concentration of 25(OH)D can be used to get a comprehensive picture of vitamin D levels from all sources. It has a half-life of two months, and it is the most accurate measure of vitamin D levels in the body [4,6].

In this study, we aimed to determine the association between vitamin D deficiency and tumor characteristics in breast cancer patients. The objectives of this study were to estimate the proportion of vitamin D deficiency among breast carcinoma patients and determine the association between vitamin D deficiency and tumor grade, histopathological subtype, and axillary lymph node status and their effect on the function of estrogen receptor (ER), progesterone receptor (PR), and human epidermal growth factor receptor 2 (Her-2/neu).

## Materials and methods

Study design

We employed a cross-sectional design to estimate the prevalence of vitamin D deficiency among breast cancer patients and its association with tumor grade, histopathological subtype, and axillary lymph node status.

Study setting

This was a hospital-based study and was conducted in the surgery department of Acharya Vinoba Bhave Rural Hospital (AVBRH), Sawangi, Wardha, Maharashtra, which is a tertiary healthcare facility. It is a 1,500-bed hospital in central India with daily general surgery patients of approximately 180-220.

Study duration

This study was conducted for two years from October 2019 to December 2021.

Sample size and sampling technique

The total sample taken for this study is 50. We used universal sampling and included all the patients (50) who were newly diagnosed with breast carcinoma based on clinical examination, radiological investigations, and histopathological confirmation. They underwent surgery for the same during the study period in this tertiary care center.

Study population

All patients newly diagnosed with breast carcinoma and who received primary surgery and pathological confirmation in the study setting were included in the study. Furthermore, patients with insufficient histopathological reports, those unfit for surgery, and those with hepatic or renal failure, metabolic bone disease, malabsorption, or recent consumption of vitamin D (patients who had received oral vitamin D in the preceding two weeks, or vitamin D injection in the preceding six months) were excluded from the study.

Study tool

After a thorough literature search, the study tool was prepared. It included three sections, namely, sociodemographic details, different characteristics of breast cancer (including various parameters), and vitamin D level (ng/mL). The prefinal version of this tool was piloted for five participants and then the final version of the tool was developed. The ultimate version of the questionnaire underwent face validation with the help of faculties and senior residents of the Surgery Department of our Institute.

Data collection

Participants fitting the inclusion criteria were included in the study. Informed written consent was taken before data collection.

Data analysis

The data were entered in Microsoft Excel, and analysis was done with the Statistical Product and Service Solutions (SPSS, version 21.0; IBM SPSS Statistics for Windows, Armonk, NY). The categorical variables were reported as frequency and percentages. Furthermore, continuous data were presented as the mean and standard deviation and median and interquartile range depending on the distribution of data. An independent t-test was used for comparing continuous variables. Categorical variables were compared using Fisher’s exact test since at least one cell had an expected value of less than five. For statistical significance, a P value of less than 0.05 was considered statistically significant.

Ethical approval

This study was conducted after approval from the Ethics Committee Department of Medical Education, Jawaharlal Nehru Medical College (Deemed to be University), Sawangi (Meghe). Data collection was not done without written informed consent. The subjects were informed about the research and procedures through a participant information sheet. The confidentiality of information has been maintained. Personal identifiers were removed during data analysis and reporting.

## Results

The mean age of the participants in our study was found to be 51.22 ± 13.7 years. Out of the 50 participants, the majority (18, 36%) of the participants were in the age group of 41-50 years, followed by 12 (24%) above 60 years, nine (18%) between 51 and 60 years, and four (8%) between 21 and 30 years.

Table 1 shows that the left breast was involved in more than half of the participants (27, 54%). The mean vitamin D level in the participants was 23.54 ± 5.53 ng/mL. Furthermore, 43 study participants (86%) showed a deficiency of vitamin D (level <30 ng/mL), and seven (14%) participants showed sufficient vitamin D levels (>30 ng/mL). As far as tumor differentiation, 40% (20) of the participants showed poorly differentiated, followed by 38% (19) who showed moderately differentiated, and 22% (11) showed well-differentiated tumor cells. Half (50%) of the participants were ER-positive, and 50% were ER-negative. PR status showed that 27 (54%) were PR negative, and 46% were PR positive. Her-2/neu status showed that 26 (52%) were Her-2/neu-positive, and 24 (48%) were Her-2/neu-negative. Furthermore, 14 (28%) were Her-2/neu amplified, followed by 13 (26%) for luminal A, 12 (24%) for Luminal B, and 11 (22%) showed tumor-negative breast cancer (TNBC). The median number of lymph nodes extracted was 15 (13-18), and the median positive lymph nodes were two (0-6).

**Table 1:** Characteristics of Breast Carcinoma ER: estrogen receptor; Her-2/neu: human epidermal growth factor receptor 2; PR: progesterone receptor

Breast carcinoma characteristics	Frequency	Percentage
Side involved		
Left	27	54.00%
Right	23	46.00%
Vitamin D levels(ng/mL)		
<30 (deficient)	43	86.00%
>30 (sufficient)	7	14.00%
Tumor differentiation		
Poorly differentiated	20	40.00%
Moderately differentiated	19	38.00%
Well-differentiated	11	22.00%
ER, PR, and Her-2/neu status		
ER status		
Negative	25	50.00%
Positive	25	50.00%
PR status		
Negative	27	54.00%
Positive	23	46.00%
Her-2/neu status		
Negative	24	48.00%
Positive	26	52.00%
Luminal status		
Her-2/neu amplified	14	28.00%
Luminal A	13	26.00%
Luminal B	12	24.00%
TNBC	11	22.00%

Table 2 shows the association of vitamin D status with various breast carcinoma characteristics.

**Table 2:** Association of Vitamin D Status with Breast Carcinoma Characteristics †: Fisher's exact test, *: independent t-test

	Vitamin D <30 ng/mL (deficient) (n=43)%	Vitamin D >30 ng/mL (sufficient) (n=7)%	P value
Age (years)			
21-30	3 (75%)	1 (25%)	0.763†
31-40	6 (83.33%)	1 (16.67%)	
41-50	16 (88.88%)	2 (11.12%)	
51-60	7 (77.77%)	2 (22.23%)	
>60	11 (91.66%)	1 (8.34%)	
Mean ± SD	51.49 ± 13.19	49.57 ± 17.6	0.735*
Side involved			
Left	22 (81.48%)	5 (18.52%)	0.429^†^
Right	21 (91.30%)	2 (8.70%)	
Tumor differentiation			
Poorly differentiated	20 (100%)	0 (0%)	0.011^†^
Moderately differentiated	16 (84.21%)	3 (15.79%)	
Well differentiated	7 (63.63%)	4 (36.37%)	
ER, PR, and Her-2/neu status			
ER status			
Negative	24 (96%)	1 (4%)	0.098^†^
Positive	19 (76%)	6 (24%)	
PR status			
Negative	26 (96.30%)	1 (3.70%)	0.039^†^
Positive	17 (73.90%)	6 (26.10%)	
Her-2/neu status			
Negative	22 (91.66%)	2 (8.34%)	0.420^†^
Positive	21 (80.77%)	5 (19.23%)	
Luminal status			
Her-2/neu amplified	13 (92.86%)	1 (7.14%)	0.113^†^
Luminal A	11 (84.62%)	2 (15.38%)	
Luminal B	8 (66.66%)	4 (33.34%)	
TNBC	11 (100%)	0 (0%)	
Number of lymph nodes extracted			
Mean ± SD	15.79 ± 5.5	14.29 ± 2.43	0.483^*^
Median (interquartile range)	15(13-18.5)	15(12.5-15.5)	
Number of positive lymph nodes			
Mean ± SD	4.42 ± 5.4	1.71 ± 2.36	0.201^*^
Median (interquartile range)	2(0-7)	1(0-2.5)	
Prognosis			
Bad prognosis	23 (100%)	0 (0%)	0.011^†^
Good prognosis	20 (74.07%)	7 (25.93%)	

The mean age for individuals with deficient vitamin D levels is 51.49 ± 13.19, whereas the mean age for individuals with sufficient vitamin D levels is 49.57 ± 17.6, and there is no statistically significant difference between them (p = 0.735). The percentage of individuals with deficient vitamin D levels tends to be higher across all age groups (>60 years), and this difference is not statistically significant. As for the side involved, there is no significant association between vitamin D status (deficient vs. sufficient) and the side involved (left or right) (p = 0.429). As far as tumor differentiation is concerned, 20 (100%) were poorly differentiated, 16 (84.21%) were moderately differentiated, and seven (63.63%) were well differentiated in the vitamin D-deficient category, and the association between vitamin D status and tumor differentiation is statistically significant (p = 0.011).

There was a statistically significant difference between vitamin D status and PR status, with deficient individuals observed to have more negative PR status compared to those with sufficient vitamin D levels (p = 0.039). However, no significant associations were observed between vitamin D status and ER status (p = 0.098) or Her-2/neu status (p = 0.420).

The mean number of lymph node-positive participants was 4.42, and the mean number of lymph nodes extracted was 15.79 in vitamin D-deficient breast cancer patients. There was a significant association between vitamin D deficiency and tumors with poor prognostic features (p = 0.11).

## Discussion

Vitamin D deficiency is common and represents a major health problem. Vitamin D is created when ultraviolet B light from the sun strikes a precursor molecule in the skin and only a small quantity of it is obtained from food. The most physiologically active form and natural ligand for vitamin D receptors is 1, 25-dihydroxyvitamin-D [1, 25-(OH) 2D]. Other target tissues that produce the activating enzyme (1 hydroxylase) and vitamin D receptors (VDRs), such as the colon, prostate, and breast, activate vitamin D outside of the kidneys to regulate cell turnover locally [4]. Early in life, vitamin D deficiency causes growth retardation and rickets, whereas in adults, it contributes to osteopenia/osteoporosis and various chronic illnesses, including autoimmune diseases, infectious diseases, and cardiovascular diseases [7-9]. Remarkably, an association between the risk of developing cancer, latitude, low sun exposure, and poor vitamin D status has been observed [4,10,11]. Vitamin D has a wide range of immunogenic and antiproliferative actions throughout the body, in addition to its well-known endocrine actions. Vitamin D deficiency has been correlated with an increased incidence of malignancies of the breast, prostate, and colon [10]. The present cross-sectional analytical study was designed to determine the correlation between vitamin D deficiency and tumor characteristics in breast cancer patients. Based on the inclusion and exclusion criteria, a total of 50 patients were included in our study.

Vitamin D levels

For a healthy Indian population, the reference range of 25-hydroxycholecalciferol (25-HCC) is much lower, and the lower limit of normal is approximately 13.5 ng/mL. This study indicates that vitamin D insufficiency in this population starts at 25-HCC values of 13.5 ng/mL and deficiency at 7 ng/mL [12]. In the present study, the majority of the participants, that is, 43 (86%), showed deficiency in vitamin D levels <30, and seven (14%) participants showed a level more than 30 with a mean level of 23.54 ± 5.53 and ranges of 7.9-36.2 (Table 1). In Yao et al.’s study on the association of Serum Level of vitamin D at diagnosis with breast cancer survival, a case-cohort analysis in the pathways study, “high” 25(OH)D levels (>30 ng/mL) were observed in 647 patients (35.9%), while 570 patients (31.7%) showed “intermediate” 25-hydroxyvitamin D (25OHD) levels (20-30 ng/mL), and 583 patients (32.4%) were classified in the “low” 25(OH)D group (<20 ng/mL) [13].

Age

In the present study, most of the participants, that is, 18 (26%), were in the age group 41-50 years, followed by 12 (24%) aged above 60 years, then 18% between 51 and 60 years. The lowest number of participants, four (8%), were in the age group 70-79 years. The mean age of the participants was 60 years, with a 13.7 standard deviation and a range of 21-84 years. While applying the association of age with vitamin D deficiency, it was observed that most, that is, 16 (37.21%), participants with vitamin D deficiency were in the age group 41-50 years, followed by 11 (25.58%) participants with vitamin D deficiency who were more than 60 years. The mean age of the vitamin D-deficient participants was 51.49 ± 13.19 standard deviation with a P value of -0.763 (not significant). In Hatse et al.’s study on vitamin D status at breast cancer diagnosis and its correlation with tumor characteristics, disease outcome, and genetic determinants of vitamin D insufficiency, a total of 1,800 eligible patients were included, and the mean age of the participants was 57.7 (22.0-94.0), which is almost similar to our study findings, 61.4 (28.0-94.0) with low 25OHD (20 ng/mL), 56.9 (22.0-93.0) with intermediate 25OHD (20-30 ng/mL), and 55.0 (26.0-88.0) with high 25OHD (>30 ng/mL) [14].

Side involved

In the present study, the most common side involved was left with 27/50 (54%) participants, and 23/50(46%) were right-sided. Most (22, 51.16%) participants with left-side involvement showed vitamin D deficiency, and 21 (48.84%) with right-side involvement showed vitamin D deficiency (P value: -0.43 nonsignificant).

Tumor differentiation

The participants were categorized based on tumor differentiation. Almost half of the participants (20/50, 40%) were poorly differentiated, followed by 19/50 (38%) who were moderately differentiated and 11/50 (22%) who were well differentiated. Most of the patients who were poorly differentiated were vitamin D deficient, 16 (37.21%) who were vitamin D deficient were moderately differentiated, and seven (16.28%) were well differentiated with a P value of -0.011, which is significant. In Huss et al.’s breast cancer research on vitamin D receptor expression in invasive breast tumors and breast cancer survival, VDR expression was evaluated in a tissue microarray of 718 invasive breast tumors. Covariation between VDR expression and established prognostic factors for breast cancer was analyzed as well as associations between VDR expression and breast cancer mortality. Most tumors, that is, 624 (91.9%), expressed cytoplasmic VDR in a high fraction (76-100%) of cells. There was a wider distribution of intensity: no stain (n = 7, 1.0%), low intensity (n = 26, 3.6%), moderate intensity (n = 174, 24.2%), and high intensity (n = 472, 65.7%) [15].

ER, PR, and Her-2/neu status

ER, PR, and Her-2/neu status of the participants were investigated, and it was found that out of 50 participants, 25 (50%) were ER-positive, 23/50 (46%) were PR positive, 26 (52%) were Her-2/neu-positive, and 24 (48%) were Her-2/neu-negative. Out of the 25 ER-positive participants, 19 (44.19%) were vitamin D deficient, 17/23 (39.53%) participants with vitamin D deficiency were PR positive, and 21/26 (48.84%) with vitamin D deficiency were Her-2/neu-positive. The P value is not significant.

Luminal status

In the present study, based on luminal status, the majority (14, 28%) were Her-2/neu amplified (13, 26%), and 12 (24%) cases were luminal A and luminal B, respectively, and 11 (22%) showed TNBC. The majority (13, 30.23%) of the participants with Her-2/neu amplified were vitamin D deficient, followed by 11 (25.58%) with luminal A, and TNBC showed vitamin D deficiency in eight (18.60%). Luminal B showed vitamin D deficiency with a P value of 0.113 (significant). When molecular subtypes were compared, it was noted that only 6.6% of luminal A-like tumors had a negative VDR expression in the nuclei as compared to 25.6% among luminal B-like tumors, and 78.4% among triple-negative tumors [15].

Lymph nodes and lymph nodes extracted

On examination of lymph nodes, the mean lymph node-positive participants were 4.04 ± 5.16 standard deviation, and the mean number of lymph nodes extracted was 15.58 ± 5.19 standard deviation. The mean of positive lymph nodes was 4.42 ± 5.4 in vitamin D-deficient participants and 1.71 ± 2.36 in vitamin D-sufficient participants with a 0.201 P value (not significant), and the mean number of lymph nodes extracted was 15.79 ± 5.5 in vitamin D-deficient and 14.29 ± 2.43 in vitamin D-sufficient participants with a P value of 0.48 (nonsignificant).

Overall prognosis

The majority (23/43, 53.49%) showed a bad prognosis, and 20/43 (20%) showed a good prognosis in vitamin D-deficient participants. No bad prognosis was observed in vitamin D-sufficient participants with a P value of 0.011, which is significant. Out of 50 participants, 43 were vitamin D deficient, and seven were vitamin D sufficient. Out of 43 vitamin D-deficient participants, almost half (20, 46.51%) were poorly differentiated and 16 (37.21%) were moderately differentiated, and 19 (44.19%) and 17 (39.53%) showed ER- and PR-positive status, while 21 (48.84%) were Her-2/neu-positive. In Huss et al.’s breast cancer research on vitamin D receptor expression in invasive breast tumors and breast cancer survival, VDR expression was found to be associated with favorable prognostic characteristics, such as small size, low grade, ER positivity, PR positivity, low Ki-67 expression, and luminal-like molecular subtypes. This corresponds to the finding that VDR-positive tumors were found to be associated with a decreased risk of breast cancer-specific mortality, but this association was also independent of other prognostic factors [15]. Goodwin et al. reported that vitamin D levels were significantly lower in women with high-grade breast tumors [5]. Conversely, Yao et al. recently found that reduced 25(O)HD levels were correlated with higher tumor grade and ER-negative tumors among premenopausal women only, but not when pre- and postmenopausal women were considered together [13]. In addition to breast cancer risk, vitamin D has also been shown to be inversely associated with breast cancer stage, recurrence, and mortality. Among a cohort of women with early breast cancer, vitamin D deficiency (defined as 25(OH)D levels <50 nmol/L versus >72 nmol/L) was associated with increased risk of both distant recurrence (hazard ratio (HR) = 1.71; 95% CI: 1.02-2.86) and death (HR = 1.60; 95% CI: 0.96-2.64) [5].

Limitations

Because the study was conducted only in one tertiary healthcare center in Central India and the small sample size because of less patient flow during the COVID-19 pandemic, the generalizability of the findings to other regions or settings may be limited.

## Conclusions

In the present study, we found that most breast cancer patients (86%) were vitamin D deficient. In this study, we also found a significant association between vitamin D deficiency and tumor differentiation and that the left side was more involved than the right side in vitamin D-deficient patients. Low vitamin D levels were shown to be a risk factor for PR along with positive lymph node status in breast cancer patients. Hence, this study provides important insights that vitamin D has a potential role in the prevention of breast cancer, it may reduce its aggressiveness, and its deficiency is associated with increased risk of breast cancer and highlights the need for further large-scale research in this field.
